# Manganese-Oxidizing Antarctic Bacteria (Mn-Oxb) Release Reactive Oxygen Species (ROS) as Secondary Mn(II) Oxidation Mechanisms to Avoid Toxicity

**DOI:** 10.3390/biology10101004

**Published:** 2021-10-06

**Authors:** Ignacio Jofré, Francisco Matus, Daniela Mendoza, Francisco Nájera, Carolina Merino

**Affiliations:** 1 Laboratory of Conservation and Dynamics of Volcanic Soils, Department of Chemical Sciences and Natural Resources, Faculty of Engineering and Sciences, Universidad de La Frontera, Avenida Francisco Salazar 01145, Temuco 4811230, Chile; ignacio.jofre@ufrontera.cl (I.J.); francisco.matus@ufrontera.cl (F.M.); daniela.mendoza@ufrontera.cl (D.M.); fnajera@uchile.cl (F.N.); 2 Network for Extreme Environment Research, Universidad de La Frontera, Avenida Francisco Salazar 01145, Temuco 4811230, Chile; 3 Department of Chemical Sciences and Natural Resources, Universidad de La Frontera, Avenida Francisco Salazar 01145, Temuco 4811230, Chile; 4 Agronomical Science Faculty, Universidad de Chile, Sta. Rosa 11315, La Pintana, Santiago 8820808, Chile; 5 Scientific and Technological Bioresources Nucleus, BIOREN, Universidad de La Frontera, Avenida Francisco Salazar 01145, Temuco 4811230, Chile

**Keywords:** ROS, Mn oxidation, Antarctic soil

## Abstract

**Simple Summary:**

Manganese (Mn)-oxidizing bacteria (MnOxb) are an essential group of microorganisms that oxidize soluble Mn(II) to form precipitate Mn(III) minerals, playing a crucial role in soil formation. The Fildes Peninsula is one of the fastest-warming areas globally and, therefore, the maritime Antarctic soils from this pivotal location allow for the examination of the effect of temperature on bacterial communities. The temperature causes an increase in the microbial respiratory rate, producing reactive oxygen species (ROS), which are harmful to bacteria. We evaluate an evasive secondary non-enzymatic mechanism for ROS production under increasing temperature in MnOxb isolated from Antarctic soils. Bacteria produce ROS capable of oxidizing Mn(II) as temperature increases, contributing to the enzymatic pathway protecting microbial cells from Mn(II) toxicity. In addition, we determine that certain strains, such as *Arthobacter oxydans*, can use these ROS as mechanisms to protect themselves from Mn toxicity at high concentrations. In conclusion, we describe a secondary mechanism of Mn(II) oxidation in bacterial strains of Antarctic soils.

**Abstract:**

Manganese (Mn) oxidation is performed through oxidative Mn-oxidizing bacteria (MnOxb) as the main bio-weathering mechanism for Mn(III/IV) deposits during soil formation. However, with an increase in temperature, the respiration rate also increases, producing Reactive Oxygen Species (ROS) as by-products, which are harmful to microbial cells. We hypothesize that bacterial ROS oxidize Mn(II) to Mn(III/IV) as a secondary non-enzymatic temperature-dependent mechanism for cell protection. Fourteen MnOxb were isolated from Antarctic soils under the global warming effect, and peroxidase (PO) activity, ROS, and Mn(III/IV) production were evaluated for 120 h of incubation at 4 °C, 15 °C, and 30 °C. ROS contributions to Mn oxidation were evaluated in *Arthrobacter oxydans* under antioxidant (Trolox) and ROS-stimulated (menadione) conditions. The Mn(III/IV) concentration increased with temperature and positively correlated with ROS production. ROS scavenging with Trolox depleted the Mn oxidation, and ROS-stimulant increased the Mn precipitation in *A. oxydans*. Increasing the Mn(II) concentration caused a reduction in the membrane potential and bacterial viability, which resulted in Mn precipitation on the bacteria surface. In conclusion, bacterial ROS production serves as a complementary non-enzymatic temperature-dependent mechanism for Mn(II) oxidation as a response in warming environments.

## 1. Introduction

Manganese (Mn) oxides are ubiquitous in the environment, being found, for example, in the form of coatings and nodules in soils, freshwater, and marine sediments and as rock varnishes in temperate, arid, and polar areas [1]. In nature, Mn can be found as Mn(II), Mn(III), and Mn(IV), and oxides participate in cycling under redox reactions in extreme environmental conditions [2]. The biogenic weathering of manganese occurs by abiotic and biotic processes; microbial oxidation plays a dominant role in Mn(II) oxidation, which leads to the precipitation of Mn oxides in the natural environment [3]. The Mn oxyhydroxide biogenesis is mediated enzymatically by MnOxb due to being energetically favorable and much faster than abiotic catalysis on the mineral surface of soils.

Most Mn oxidation studies have been carried out in aquatic ecosystems, not in the soil environment. As Mn oxidation occurs, insoluble Mn is precipitated on the surface of bacteria; presumably, this result is thermodynamically favorable and would create terminal electron acceptors for bacterial respiration [4], in turn protecting the bacteria cells from harsh environmental conditions [5]. The biological Mn oxidative process begins in the stationary phase, in the outer surface of bacterial cells, and is performed by multicopper oxidases (MCO) as PO [6]. However, the physiological function of bacterial Mn(II) oxidation remains unclear [7].

The processes of biological adaptation to global warming in the Antarctic environment have resulted in changes in microbial soil diversity [8,9], inducing pedogenic dynamics in the soil structure, soil organic matter (SOM), and nutrients [10]. The side effects of increasing temperatures have been observed in microbial respiration in Antarctic soils, the sub-Antarctic Southern Ocean [11], and Arctic sediment [12], where warming enhances the aerobic respiratory cycles of microorganisms. Studies have demonstrated that the temperature is crucial for the bacteria to oxidize Mn(II) to Mn(III) or Mn(IV) using enzymatic pathways; however, temperature could be controlled for different molecular mechanisms to command the oxidization of Mn(II) [13]. The significant influence of temperature has been reported in the production of metabolic by-products, such as radicals and ROS in aerobic [14], microaerobic, and anaerobic bacteria [15]. ROS perform regulatory functions for metabolism and control important biofilm formation activities [16,17]. Although the mechanisms of ROS production in bacteria are not clear, it has been hypothesized that membrane NADPH oxidases and electrons are released from the Q-cycle of the cytochrome bc1 complex [18], serving as sources.

**Figure 1:**
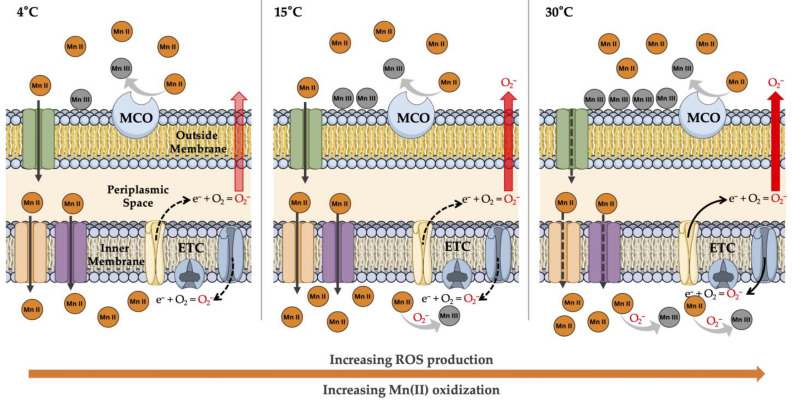
Schematic diagram of the hypothetical activity of ROS-mediated Mn(II) oxidation. Multicopper oxidases (MCO), the enzymatic oxidative pathway, generate constitutive Mn(II) oxidation outside the membrane and precipitate as Mn(III). At warming conditions (up to 4 °C), electrons (e^−^) are released (solid arrow) from the electron transport chain (ETC), reacting with O_2_, forming superoxide anion (O_2_^−^). As temperature increases, the permeable Mn(II) by Mn transport is accumulated in intracellular milieu, inducing a toxic effect; thus, the Mn channels reduce the transport (dashed arrow). The ROS produced via ETC (inside) react with intracellular Mn(II) or are diffused through the membranes, reacting with Mn(II) in extracellular milieu and deposited as oxides. This mechanism could be an indirect mechanism of Mn(II) oxidation in a pathway unrelated to MCO.

ROS include the superoxide anion (O_2_^−^), a relatively non-reactive species that stimulates the successive production of hydrogen peroxide (H_2_O_2_) by superoxide dismutase (SOD) as an initiator to produce the most oxidant-reactive species: hydroxyl radical (OH^−^) and anions (OH). Although ROS are usually regarded as deleterious agents for bacteria, a new role for ROS has been proposed in the weathering of Mn(II) in marine surface waters. The study of Learman, et al. [19] presented the first empirical evidence on the formation of manganese oxides by superoxides in seawater. This was later demonstrated in marine *Roseobacter* sp. by producing a strong and versatile redox reactant superoxide [20], supporting the dark bacterial production of superoxides in metal cycling and biogenic mineralization in aquatic environments. However, few studies have investigated the mineral oxidation processes through ROS production pathways in soil bacteria [21]. These studies indicate evasive mechanisms of aluminum toxicity by H_2_O_2_ production in *Pseudomonas fluorescence* [21] and endogenous radicals as detected in Antarctic bacterium [22]. Accordingly, we assume that increasing respiration of Antarctic MnOxb under global warming can induce similar evasive mechanisms as mediated by Mn oxidation. We hypothesize that MnOxb can produce and release ROS as a secondary temperature-dependent mechanism of Mn(II) oxidation as a non-enzymatic process of Mn(III/IV) deposits (Figure 1). Soluble Mn(II) is oxidized by extracellular MCO and PO activity as constitutive mechanisms of metal oxidation. As temperature increases, the Mn(II) gradient turns into a detrimental effect, and the Mn channels begin to close to limit the mobilization of Mn(II). In addition, the respiration rate increases, and the release of electrons from the electron transport chain (ETC) increases the reactivity with O_2_, forming O_2_^−^, which can oxidize Mn(II) inside or diffuse through the membranes as H_2_O_2_ and/or OH^−^, reacting with extracellular Mn(II). To test our hypothesis, we evaluated, in fourteen MnOxb strains from coastal Antarctic soils, ROS production and Mn(II) oxidation under increasing temperatures as a secondary mechanism of biogenic Mn weathering. The objective of this study was to provide insight into the biogeochemical mechanisms of metal weathering in Antarctic soils by native bacteria, induced by climate change.

## 2. Materials and Methods

### 2.1. Soil Sampling

Soil samples were obtained from the top mineral layer (5–15 cm) of the Fildes Peninsula (62°10′21′′ S/58°55′13′′ W; January 2019) described as Entisol, Inceptisol, and Ornitosol classification (Soil Survey Staff, 2014) developed under the vascular plants *Deschampsia antarctica* and *Colobanthus quitensis*. Site location elevation 5–30 m.a.s.l., mean annual temperature 0 °C, and precipitation 493 mm were obtained using a combination of global positioning system (GPS), geographic information system (GIS), and site digital elevation models. The study was conducted at ten sites located at increasing along-shore distances from Collins Glacier. After removing the organic horizons, four composite soil samples from each site (*n* = 40) were extracted from the top mineral horizon and transported to the laboratory under cold conditions. In the laboratory, the samples were cleaned of coarse organic debris and separated into two portions: one portion was stored at 4 °C for microbial and enzymatic analyses, and the second was air-dried for further physicochemical analyses. Physical variables were evaluated by sieving soil particles larger than 2 mm. The pH was measured in a 1:2.5 soil:water suspension. Soil C was determined using TOC-VCSH (Shimadzu, Kyoto, Japan), and total N was determined by Kjeldahl distillation (VELP, Usmate, Italy). Other soil properties, such as active (Mn), were extracted using 0.2 M ammonium oxalate at pH 3 (showed as Mn_o_) [23]. We also performed an extraction to identify the exchangeable, crystalline, and complexed-SOM Mn using dithionite-citrate-bicarbonate (Mn_d_) [24]. The Mn concentrations were determined by atomic absorption spectroscopy (Perkin Elmer 3110, Waltham, MA, USA) using an air/acetylene flame at 279.5 nm (Table S1).

### 2.2. Bacterial Isolation and Identification

One gram of each soil sample was serially diluted (10^−1^ to 10^−8^) and plated (0.1 mL) on two different culture media: (1) Plate Count Agar (PCA, per liter; 5.0 g enzymatic digest of casein, 2.5 g yeast extract, 1.0 g glucose, and 15 g agar, pH 7.2) (Difco, Becton Dickinson Sparks, MD, USA) and (2) Mn(II)-agar, consisting of the following reagents per liter; 10 mmol L^−1^ HEPES buffer (pH 7.4), 0.001 g FeSO_4_ × 7H_2_O, 2 g peptone (Becton Dickinson, Sparks, MD, USA), 0.5 g yeast extract (Becton Dickinson, Sparks, MD, USA), 15 g agar, and 0.30 g MnSO_4_ × H_2_O as Mn(II) source (in total 1982 µmol/L Mn(II)). For both, samples were cultured at 15 °C. The total content of psychrophilic aerobic bacteria was counted in PCA. The colonies that presented a black coloration or brown (Mn(III) [or Mn(IV)] precipitates) in Mn(II)-agar were counted as MnOxb [25,26]. All samples were counted as colony-forming units per g of soil (CFU/g).

### 2.3. Bacterial Identification by MALDITOF-TOF

Bacterial identification at the species level was performed by Matrix-assisted laser desorption ionization-time of flight mass spectrometry (MALDI-TOF/MS) and database (Maldi-Biotyper; Bruker Daltonics, Bremen, Germany) comparison. This technique was selected for its speed and precision in determining taxonomic groups according to the comparison of spectra with a database with known and identified bacteria. After MnOxb was cultured in Nutrient Broth (NB, per liter; 1 g beef extract, 2 g yeast extract, 5 g peptone, 5 g NaCl, pH 7.0), the samples were centrifuged, washed twice in purified water, and fixed overnight with ethanol (70%). Then, samples were centrifuged (10,000× *g*) and the resultant pellets were dried at room temperature. The protein extraction was performed as described by Dingle and Butler-Wu [27] with acetonitrile (ACN, Merck Milipore, Darmstadt, Germany)-formic acid (FA, Merck Milipore, Darmstadt, Germany). Two microliters of the supernatant were mounted on MALDITOF-TOF MS Target Plate covered with saturated solution of 4-hydroxi-cinamic acid diluted in 50% ACN with 2% trifluoroacetic acid (Sigma Aldrich, Milwaukee, WI, USA). The mass spectra, obtained with MALDITOF-TOF MS-Autoflex Speed (Bruker Daltonics, Bremen, Germany), were compared in the microbial library through MALDI Biotyper Compass 4.1 software (Bruker Daltonics, Bremen, Germany). Strains with a match score > 2.0 were identified at the species level. For this study, only the strains identified with a different species were selected and worked. In total, 14 different strains were studied.

### 2.4. Inoculum Preparation and Temperature Experiment

The biogenic Mn(II) oxidization was evaluated under 3 temperatures of incubation—4 °C, 15 °C, and 30 °C—under in vitro conditions for all isolated strains. The starter inoculum for each bacterial strain was produced in 100 mL of Nutrient Broth until to the stationary phase was reached (0.99 of optical density (OD) at 600 nm). Then, 10 µL of inoculum was placed into 290 µL of Mn(II)-broth in optical 96-well reaction plates and incubated for 24, 48, 72, 96, and 120 h in an orbital shaker at 4 °C, 15 °C, and 30 °C. Each strain and temperature was evaluated on independent plates in a destructive sampling design for each time interval. In total, 630 wells were evaluated (fourteen strains, three temperatures, five incubation times, and three replicates) for enzymatic, growth, and manganese oxidation. Controls consisting of culture media without an Mn(II) source were included.

### 2.5. Quantification of Mn(III/IV) and Bacterial Growth Kinetics

The biogenic Mn(II) oxidation was evaluated using colorimetric dye leucoberbelin blue (LBB) assay, which reacts with Mn(III) and Mn(IV) [28]. From each incubation time, 300 µL of bacterial suspension was recovered, and the OD 600 nm was directly measured for kinetic growth. Then, 100 µL of bacterial suspension was added to 100 µL of LBB 0.08% (in 45 mmol/L of acetic acid) in a 1.5 mL amber tube. The mixture was vortexed and incubated for 5 min at room temperature in darkness. The resultant colored solution was mounted in optical 96-well reaction plates, and the OD at 628 nm was registered in a microplate reader (AccuReader, Metertech Inc., Taiwan, China). The data were compared with a calibration curve containing culture medium and KMn as an Mn(IV) source in a linear range from 0 to 1000 μmol/mL.

### 2.6. Peroxidase Activity and ROS Production

A new batch of culture bacteria was produced for this analysis. From the initial inoculum 30 µL of bacterial suspension was placed in 1 mL of Mn(II) broth and incubated for 120 h at 4 °C, 15 °C, and 30 °C under constant stirring. At 120 h of incubation, the PO activity and ROS production were evaluated for each temperature. The PO activity was evaluated according to Bach, et al. [29]. A total of 900 µL of bacterial suspension was taken from the solution and mixed with 60 mL of sodium acetate buffer, stirred in vortex, incubated for 2 min, and centrifuged for 10 min at 2000× *g*. The PO activity was evaluated in a 3 mL cuvette containing 1 mL MnSO_4_ (1.0 mmol/L) for oxidation, 1 mL supernatant sample, 0.5 mL H_2_O_2_ (2.0 mmol/L), and 0.5 mL of sodium tartrate buffer (20 mmol/L) at pH 4.5. The PO activity was determined spectrophotometrically at 238 nm. All oxidative enzymatic activities were expressed in millimoles (mmol/min) of substrate oxidized per minute. For ROS production, the analysis was carried out using MitoSOX Red [30]. This dye reacts with superoxides and other radical species, exerting a red fluorescence after oxidation and serving as an indicator of ROS production; its fluorescence is directly proportional to the level of ROS production. After incubation, 100 µL of bacteria suspension was centrifuged at 2000× *g* for 10 min. The supernatant was discarded and the same volume of phosphate saline buffer (PBS, Thermo Fisher Scientific, Madrid, Spain) was added. The samples were stained with MitoSOX Red (Invitrogen, Thermo Fisher, Waltham, MA, USA) at (3.5 µmol/L in 0.02% [*w*/*v*] Pluronic F127) and incubated for 15 min at 37 °C to facilitate diffusion through the membrane. At the end of incubation, the samples were immediately measured in a multimodal microplate reader (Synergy-HT, Biotek, Winooski, VT, USA) at 510 nm_ex_/580 nm_em_. PO activity and ROS production were compared with respect to bacterial culture without the Mn(II) source on broth, under the same incubation conditions.

### 2.7. Contribution of ROS Production on Mn(II) Oxidation

*Arthobacter oxydans* was the strain with the best ROS performance to oxidize Mn(II) to Mn(III) or (IV). To verify that the production of ROS from *A. oxydans* tends to oxidize Mn(II), we tested the oxidation of Mn(II) in the presence and absence of menadione (MND), a quinone that undergoes catalytic one-electron redox cycling to reduce molecular oxygen, forming O_2_^−^ [30,31,32]. Otherwise, TROLOX (6-hydroxy-2,5,7,8-tetramethylchroman-2-carboxylic acid) (0.05 mmol/L) (Vitamin E antioxidant analog) was used as an ROS scavenger to avoid the Mn(II) by the ROS pathway. The strains were incubated under four experimental conditions: (i) control, (ii) Mn(II) (1.35 mmol/L), (iii) Mn(II) (1.25 mmol/L) + MND (50 µmol/L), and (iv) Mn(II) (1.25 mmol/L) + Trolox (50 µmol/L). From the initial inoculum in NB (0.1 OD 600 nm), 100 µL was taken and cultured in 9 mL of respective liquid medium. All samples were incubated for 120 h and evaluated at intervals of 24 h in a destructive sampling design. In total, 15 cultures were evaluated (three treatments and five replicates). The ROS production and bacterial growth were evaluated.

### 2.8. Influence of Mn(II) on Membrane Potential, Viability, and Bacterial Morphology

We included the analysis of membrane potential using 3,3′-dihexyloxacarbocyanine iodide (DiOC6(3)) and Propidium Iodide (PI) as an indication of the physiological states of the cells and membrane damage [33] to evaluate the bacterial response under increased Mn(II) concentration at the temperature that best promotes the production of ROS. A total of 100 µL of initial inoculum of *A. oxydans* was cultured in 9 mL of culture medium supplemented with Mn(II): 0.15, 0.85, and 1.25 mmol/L for 120 h at 30 °C and 170 rpm. At the end of incubation, 100 µL of bacterial suspension was stained with 0.5 µmol/mL of DiOC6(3) (in DMSO) and 0.15 µmol/mL of PI, incubated for 10 min at 37 °C, and immediately evaluated in a microplate reader (Synergy HT, Biotek, Winooski, Vermont, USA) at 488 nm_ex_/520 nm_em_. All samples were compared with respect to the control without Mn(II). All evaluation was performed with 3 biological replicates. Finally, 200 µL of cultured strain was fixed in 3% glutaraldehyde in 0.1 moles of phosphate buffer at pH 7.2 for 1 h at 4 °C and washed twice; the pellet was analyzed in SEM/EDX (HITACHI) to detect changes in shape and structure.

### 2.9. Statistical Analysis

Statistical analysis was carried out in GraphPad Prism (version 6.0, GraphPad, San Diego, California, USA). Bacterial kinetic and LBB analysis were evaluated by non-lineal regression (exponential growth equation). The distribution of each non-lineal equation (exponential growth equation $Y=Y0\times exp\left(k\times X\right)$ and second-order polynomial equation Y=B0+B1×X+B2×X^2) was validated using the best fitting XY data point through Curve expert 5.0 (Hyams Developments, Chattanooga, Tennessee, USA). Pearson coefficients between ROS production, Mn oxidation, and temperature parameters were computed after normal distribution analysis. The PO activity and O_2_^−^ were tested using Tukey’s HSD. Multiple comparisons were assessed using a SIDAK test with a *p*-value of 0.05. Tests for normality were conducted before performing ANOVA, and abnormally distributed datasets were log-transformed when necessary. Levene’s test was performed to evaluate the homogeneity of variance.

## 3. Results

### 3.1. Manganese Oxidizing Bacteria in Antarctic Soils

The content of cultivable aerobic psychrophilic bacteria (APB) and MnOxb was detected in all soil samples (Table 1). Soils from colluvium parent material (S5 and S9) contained a more significant number of cultivable psychrophilic microorganisms (9.2 × 10^6^ and 8.7 × 10^6^ CFU/g). However, MnOxb was detected in soil samples derived from colluvium parent material and ornitosol (S6; 9.4 × 10^3^ and ANT-16; 6.5 × 10^3^ CFU/g), representing 6.4% and 1.2% of the total APB count. The genera *Pseudomonas* spp. (gammaproteobacteria) and *Arthrobacter* spp. (actinobacteria) were MnOxb isolated (34.2% and 21.1%) and obtained in all soil samples. Moreover, a lower content of MnOxb was detected in S1 and S3, both from cryoturbant parent material (see soil characteristics in Table S1). In total, 14 strains differing in identity at the species level were selected for further analysis.

**Figure 2:**
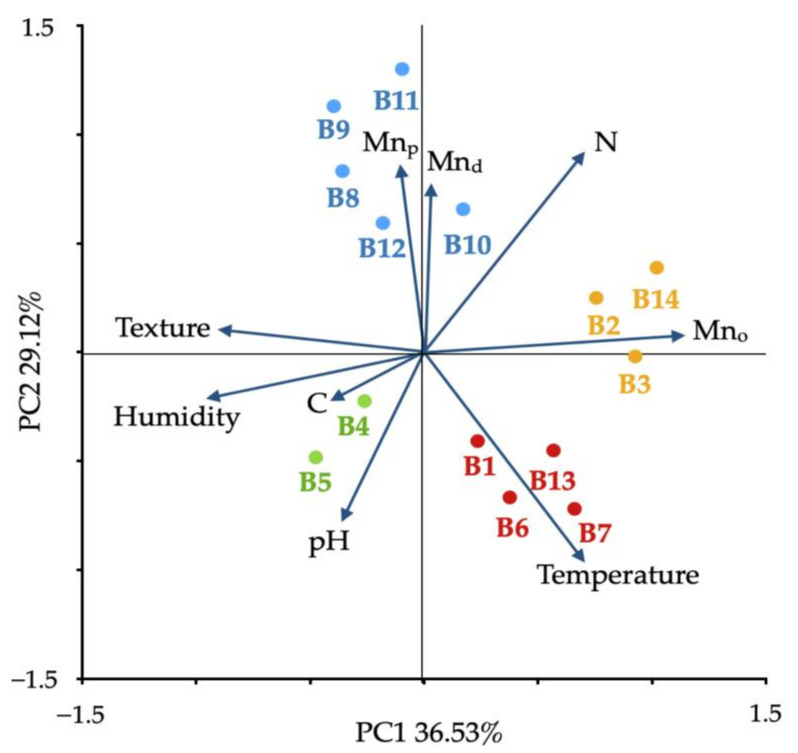
Principal component analysis (PCA) based on soil physicochemical characteristics as variables.

The results of PCA explained 66% of the total variance (PC1: 36.53% and PC2: 29.12%) (Figure 2). Based on soil factors, the PCA analysis showed that the bacteria strain formed four clusters: Group 1 (blue) contained five strains associated with the presence of pyrophosphate extractable Mn (Mn_p_) and dithionite extractable Mn (Mn_d_) in the soil; Group 2 (red) contained four strains associated with the temperature changes; Group 3 (yellow) contained three strains associated with the presence of oxalate extractable Mn (Mn_o_); and Group 4 (green) contained two strains associated with the percentage of carbon content. The other variables of soil (N, pH, humidity, and texture (clay content)) are not associated with the presence of specific strains.

### 3.2. Effect of Temperature on Bacterial Growth and Mn(II) Oxidation

**Figure 3:**
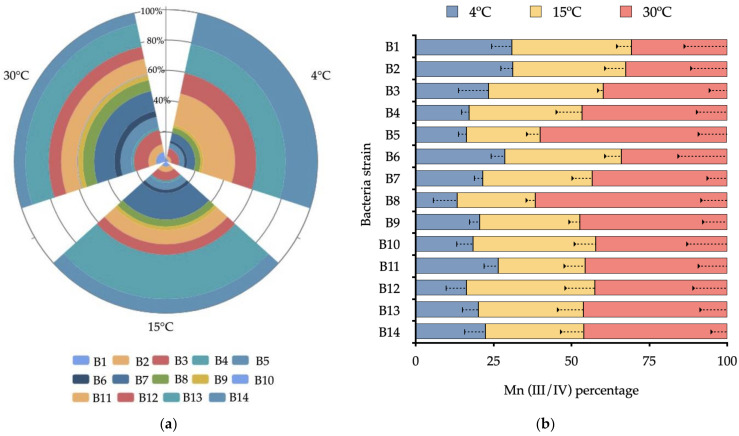
(**a**) Accumulated bacterial growth kinetics (at OD 600 nm) at 4 °C, 15 °C, and 30 °C from 24 to 120 h. (**b**) percentage of Mn(III/IV) produced from 24 to 120 h at 4 °C, 15 °C, and 30 °C.

The growth kinetics and the accumulated Mn(III/IV) of each strain were evaluated under 4 °C, 15 °C, and 30 °C for 120 h (Figure 3a). All strains were affected by temperature in Mn(II) enrichment broth. The higher increases in bacterial growth (OD600 nm) were obtained at 30 °C by B13 (15%), B7(13%), and B11(10%), reaching densities of 1.71 ± 0.03, 1.73 ± 0.05, and 1.74 ± 0.1, respectively. The same behavior was observed at 4 °C. The rate constant showed an increase of this value from 0.013 ± 0.002 to 0.027 ± 0.002 OD/h. The lowest growth density was always observed at 4 °C, where the strains B4 and B10 reached values less than 0.4 OD 600 nm (1% of the total of evaluated strains). The strain B10 did not show significant differences (*p* > 0.05) between the maximal OD obtained at 120 h after the three temperatures were evaluated.

The content of Mn(III/IV) increased during the incubation (Figure 3b), except for B9, which did not show significant differences between temperatures. Higher contents of Mn(III/IV) were achieved by B8 (61.7%) and B5 (60.2%) at 30 °C, equivalent to 1.64 ± 0.10 µmol/mL. Lower content of Mn(III/IV) at 120 h was observed in B8 (13.3%), followed by B12 (16.2%) and B4 (17.1%) at 4°C, with values of 0.25 ± 0.02, 0.29 ± 0.02, and 0.31 ± 0.02 µmol/mL, respectively. Bacterial growth and Mn(II) oxidation correlated in all strains, except for B9, which did not show significant correlation (r = 0.22) between bacterial growth and Mn(III/IV) production at 30 °C (Table S2).

### 3.3. Peroxidase Activity and ROS Production under Increased Temperature

**Figure 4:**
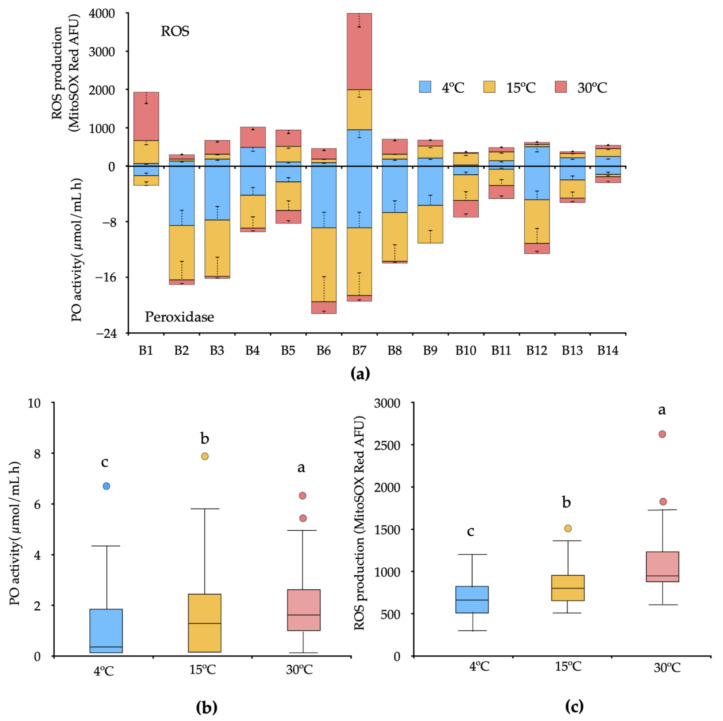
(**a**) Effect of temperature (4 °C, 15 °C, and 30 °C) on peroxidase (PO) activity and ROS (O_2_^−^) production (MitoSOX red fluorescence). Values were normalized with respect to the control (without Mn(II)). (**b**,**c**) Boxplots showing median, minimum, and maximum values; first and third quartile and outliers (colorful dots) of the PO activity and ROS production, respectivelly. Different letters show significant differences with *p* < 0.05.

The PO activity and ROS production (O_2_^−^) in each strain were evaluated in a culture medium with and without Mn(II) at 4 °C, 15 °C, and 30 °C at 120 h of incubation (Figure 4). We observed significant differences among treatments in all temperatures evaluated. The PO activity was variable between the strains, and all the strains showed reduced activity when cultured in a medium supplemented with Mn(II) as compared with PO basal activity (not shown). A higher decrease of activity was observed in the strains B6 and B7 at 4 °C and 15 °C, and a lower decrease of activity was observed in the strains B1, B14, and B12 (Figure 4a). However, the control cultures in the absence of Mn(II) always tended to increase the activity. We observed that all strains revealed ROS production at all temperatures (Figure 4a). The strain B2 (~102.22 AFU) and B10 (~121.18 AFU) were the only strains that did not increase the ROS production after Mn(II) treatment at all temperatures evaluated. After Mn(II) treatment, strains B7 (~1387.56 AFU) followed by B1 (~644.61 AFU) were the strains with high ROS production as the temperature increased. Some strains, such as B1, B5, and B10, showed significant differences in ROS production after Mn(II) treatment at 15 °C, but not at 4 °C and 30 °C, where the control level increased at the manganese level treatment.

The dispersion of bacterial PO enzyme released within each temperature was examined by measuring the distance between the centroid. Bacterial dissimilarity within each temperature was the lowest at 4 °C and the highest at 30 °C (Figure 4b). The same trend in dispersion was observed in ROS production (Figure 4c).

### 3.4. Influence of ROS Production on Mn Oxidation

**Figure 5:**
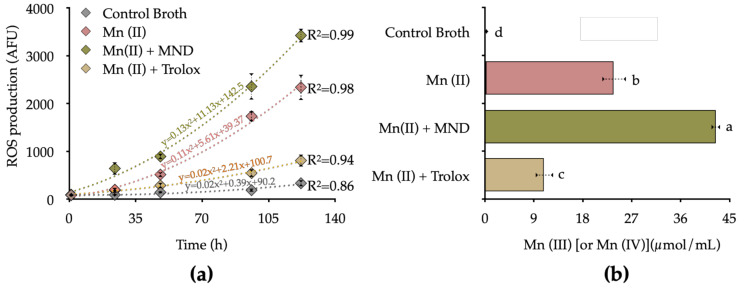
Influence of ROS production/scavenging on Manganese oxide production. (**a**) *A. oxydans* strains cultured in Mn-enriched broth (Control) supplemented with Mn(II), menadione (MND) as ROS stimulator, and Trolox as ROS scavenger at 15 °C after 120 h of incubation. (**b**) Mn(III/IV) production at 120 h of incubation. Different letters show significant differences with *p* < 0.05. Data were plotted using a second-order polynomial (quadratic) curve.

To evaluate the ROS production’s influence as a mechanism that increases Mn oxide, we focused on *Arthrobacter oxydans* (B7); this strain at temperatures over 15 °C increased significantly the levels of ROS production and Mn(II) oxidation in comparison with the control after 120 h. We evaluated the influence of ROS production on biogenic Mn(II) oxidation by inhibition and stimulation of bacterial ROS release, using the ROS-inducer menadione (MND) and the ROS scavenger Trolox (Vitamin E). The contribution of bacterial ROS production and Mn(II) oxidation are shown in Figure 5a.

The ROS inducer (MND) added in the Mn(II) enriched culture medium resulted in enhanced bacterial ROS production, reaching values 9.34 times than the control (without Mn) and 7.05 times higher than bacteria treated with Mn(II) at 120 h. Under antioxidant conditions, Trolox significantly reduced the bacterial ROS levels, reaching 0.35 times the total ROS production with respect to the Mn(II) treatment, and 1.34 times more than the control group at 120 h. This tendency was observed in Mn(II) production, were the oxidation rate rate was considerably lower in the control group (0.28 ± 0.04 µmol/mL), followed by the antioxidant condition Mn(II) + Trolox (10.94 ± 0.74 µmol/mL), Mn(II) (23.7 ± 2.18 µmol/mL), and ROS-induced condition Mn(II) + MND (42.45 ± 1.59 µmol/mL) at 120 h of incubation (Figure 5b).

### 3.5. Scanning Electron Microscopy

**Figure 6:**
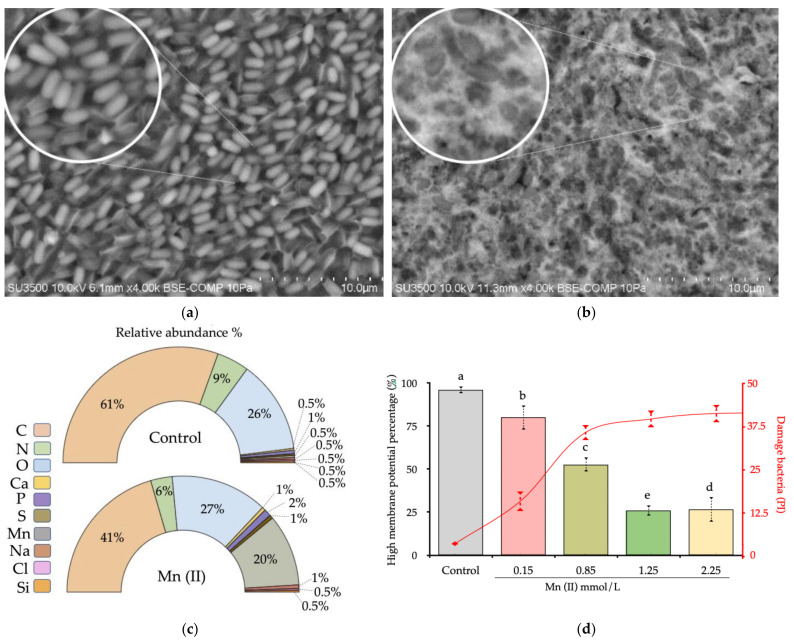
Morpho-physiological structure and membrane potential of *A. oxydans* after Mn(II) treatment. (**a**) Control. (**b**) Mn(II). (**c**) SEM-EDX analysis and comparison of detected elements between treatments. (**d**) Membrane potential by DiOc (6) fluorescence at increasing concentration of Mn(II) (each color bar) at 120 h of incubation at 30 °C. Different letters show significant differences with *p* < 0.05.

The analysis of SEM-EDX showing the *A. oxydans* isolated from the control (culture medium without Mn addition) (Figure 6a) and Mn(II) treatments (Figure 6b) indicated, after 120 h of incubation at 30 °C, a hazy coverage on the bacterial cell surface in Mn treated cells. Coupled energy dispersive X-ray fluorescence (EDX) to SEM revealed that the coverage on bacteria was precipitated Mn (on average, 24.9% ± 6.78) (Figure 6c), which was not found in the control. The membrane potential was evaluated at different Mn(II) concentrations as a physiological response to Mn(II) stimuli. We observed that Mn(II) caused a decrease of membrane potential, with increased damage in the membrane bacteria (40.3%) at high concentrations (Figure 6d). This effect was observed in all Mn concentrations.

## 4. Discussion

Our study evaluated the potential of nonconventional enzymatic mechanisms of Mn(II) oxidation by ROS production under increasing temperature conditions for bacteria isolated from ten sites in maritime Antarctic soils. A wide range of aerobic psychrophilic bacteria in the range of 2.1 × 10^5^ to 8.7 × 10^6^ UFC/g in was observed (Table 1). The higher APB count was observed in S9 and S10, associated with the presence of Mn (Figure 2), notwithstanding the differences in the available carbon content, pH, texture, and moisture (Table S1).

Significant differences in bacterial growth kinetics and Mn(II) oxidation according to temperature increases were measured for MnOxb (Figure 3). The evaluated treatments showed developing psychrophilic microorganisms in diverse temperature ranges under controlled conditions (4 °C to 30 °C). Similar effects were observed by Romanovaskaia, et al. [34] in isolated psychrophilic and mesophilic Antarctic bacteria (1 °C to 30 °C). In addition, the study revealed that 50% of the bacteria that grow in high temperatures (30 °C) show increased biomass under low temperatures (1 °C) and show dependency from biotope sources of C. In a wider range, the study of Piazza, et al. [13] observed that Antarctic terrestrial bacterial communities, as opposed to aquatic ones, have greater efficiency in range from −11 °C to 27 °C, probably because the temperature variation is high at the continental level. These studies are in agreement with our experimental conditions, where a wide variation of bacterial growth kinetics exist. The species*, A. oxydans *(B7)*, B. megaterium* (B10), *L. plantarum *(B11), and *R. fascians *(B13) grow in major abundance at 15 °C and 30 °C; however, this tendency to oxidize Mn(II) did not follow the same pattern between the evaluated strains. Furthermore, these differences were also observed in species for the same genus, such as *Arthrobacter* spp., *Rhodococcus* spp., and *Bacillus* spp., suggesting intraspecific behavior with regard to temperature tolerance (Figure 3 and Figure 4)**.** This implies that the physiological function of Mn in any of its valences can fulfill different roles and that it is closely associated with the bacterial genus and the environment source, effects that have been demonstrated in biological Mn filtration systems in wastewater, where the bacteria showed intraspecific variations around the Mn(II) oxidizing activity [4,13].

The redox transformations of Mn in the environment are directly associated with microorganisms because abiotic reactions have kinetic limitations for their transformation. However, very few microorganisms are known to have a metabolism based solely on Mn oxidation [35], unlike the autotrophs of iron (Fe II) and sulfur [36]. The MCO (*Bacillus* sp. SG-1 [37,38,39], *Pseudomonas putida* GB-1 [40,41], and *Leptothrix discophora* SS-1 [42,43]) and cyclooxygenases/peroxidases (*Erythrobacter* sp SD21 [44], *Aurantimonas manganoxydans* SI85-9A1 [45], and *Pseudomonas putida* GB-1 [46]) have been identified as responsible for the direct oxidation of Mn(II) through single electron transfer steps to form an intermediate of Mn(III). Our study evidenced that all microorganisms produce PO enzymes, a type of multicopper oxidase; however, at a high concentration of Mn(II) (108.9 mg/L), the enzymatic activity was significantly lower in all strains. The main factor that affects the PO enzymatic activity is a decrease in temperature (Figure 4a). The most significant decrease in catalytic activity was observed at 4 °C and 15 °C; this is because the optimal PO activity is over 30 °C, according to the study carried out by Alvarez, et al. [47], suggesting that decreased temperature could have a counteracting negative effect on enzyme pools in soils.

From the first reports that indicate ROS’s physiological function as a metal oxidation mechanism [48], new hypotheses were put forth on the geochemical importance of the radical species in bacteria, in contrast to the already-known deleterious effects. Our experiments showed that ROS production is time-dependent on manganese-oxidizing microorganisms, increasing the Mn(II) to Mn(III/IV) transformation (Figure 4). This new evidence suggests that soil microorganisms can produce radical species and oxidize manganese by temperature variations (Figure 4c). It is important to indicate that the fluorometric methods used in this study act as intracellular O_2_^−^ sensors (MitoSOX red); consequently, our approach demonstrates at least the endogenous ROS production in response to exogenous Mn(II). Learman, Voelker, Vazquez-Rodriguez and Hansel [20] demonstrated the concomitant O_2_^−^ production and indirect Mn(II) oxidation in *Roseobacter* AzwK-3b, data that supported the formation of oxidative radicals by the extracellular NADPH oxidase pathway in isolated bacteria from marine sediment and then detected in the ecological and phylogenetic diversity of heterotrophic bacteria [49]. However, the role of intracellular ROS is directly associated with processes that are harmful to bacterial physiology rather than with biogenic weathering activity. In addition, psychrophilic bacteria have developed protection systems against oxidative stress in low-temperature environments [50], especially due to the effect of UV radiation (photofenton) [51], increasing the levels of radical species, and in response, creating a large amount of antioxidant enzymes such as catalases, superoxide dismutases (FeSOD and MnSOD) [52], and glutathione to avoid the oxidative damage [53].

We detected a positive significant correlation between ROS production rate and Mn(III/IV) content as reflected by the increasing the temperature of the culture (Figure S1). This effect is mainly given in *Arthrobacter oxydans*. Genetic studies show that *A. oxydans* possesses an important battery of putative genes that encode oxidases, detoxifying enzymes that generate H_2_O_2_ and possibly O_2_^−^ as a by-product [54]. Increasing the temperature can enhance the generating activity of these uninhabitable by-products of aerobic respiration [55]. In addition, some metabolites of soil redox activity can promote a greater production of these molecules in soil bacteria [22,56]. On the other hand, the respiratory rate in direct relation to the function of the ETC suggests similar mechanisms in the production of intracellular ROS between bacteria and mitochondria; thus, the release of electrons (2–5%) from complex III from ETC can presumably generate O_2_^−^ in an oxygen-rich environment, a well-studied mechanism in mitochondria and a suggested mechanism for bacteria due to the similar biochemical mechanisms [57,58]. When evaluating the effect of menadione, a cytochrome C releaser that promotes the formation of O_2_^−^ by redox cycling [59], a substantial effect of Mn(III/IV) production was observed in *A. oxydans* culture while under antioxidant treatment with Trolox; the production of Mn(III/IV) was reduced (Figure 5b). The production of ROS from *A. oxydans* can be promoted to reduce the toxic effects of highly soluble Mn(II) [60], protect the cytoplasmic content, and to compensate the load of radicals [61]. Through bacterium’s enzymatic antioxidant regulators, such as superoxide dismutase and catalase. In this sense, the reduction of the membrane potential is a clear effect of avoiding the exposure of extracellular elements that can affect bacterial viability [62] (Figure 6d). This reduction of the potential is accompanied by Mn deposits in the polysaccharide matrix on the bacterial surface (Figure 6). Here, Mn serves as an ROS scavenger in physiological conditions where a low activity of antioxidant enzymes serves as a non-enzymatic mechanism of regulation of radical species in polar environments. This hypothesis is in accordance with the studies of structure-function of Mn exporters (MntP), characterized by the exclusion of Mn surplus from the intracellular milieu; where there is overexpression of this transporter, the ROS levels exceed toxicity thresholds [63]. Therefore, our hypothesis opens an important dialogue on the role of manganese in the oxidative detoxification processes as a mechanism of adaptation to the cold in the formation of the Antarctic soil.

## 5. Conclusions

Manganese-oxidizing microbial communities are critical biotic agents of the biogenic processes of soil formation, acting through intrinsic mechanisms. They are widely distributed in the prokaryotic kingdom, especially in Antarctic regions where changes in the soil are subject to climatic variations. However, many biochemical and physiological processes are not well understood. Here we reviewed the oxidative function of extracellular enzymatic mechanisms with respect complementary mechanisms for Mn oxidation in a reduced environment. Our research yields relevant results associated with the intracellular production of ROS as an important activity in the precipitation of Mn(II) in soils. It opens new hypotheses about the role of ROS-producing bacteria in abiotic processes linked to the geochemical cycles promoted by climate change.

## Figures and Tables

**Table 1:** Species detected by MALDITOF-TOF MS (BIOTYPER databank) from cultured bacteria in Mn-Ox agar.

SITE	APB ^1^	MnOxb ^2^	Strain Code	Closest Related Species	Class	log (Score) ^3^
S1	5 × 10^5^ ± 0.48	2 × 10^2^ ± 0.73	B1	*Microbacterium esteraromaticum*	Actinobacteria	2.34
S2	2.1 × 10^5^ ± 0.35	2.3 × 10^2^ ± 0.89	B2	*Pseudomonas extremorientalis*	Gammaproteobacteria	2.17
S2	2.1 × 10^5^ ± 0.35	2.3 × 10^2^ ± 0.89	B3	*Variovorax paradoxus*	Betaproteobacteria	2.15
S3	4.4 × 10^5^ ± 1.18	2.2 × 10^2^ ± 0.27	B4	*Arthrobacter psychrolactophilus*	Actinobacteria	2.09
S3	4.4 × 10^5^ ± 1.18	2.2 × 10^2^ ± 0.27	B5	*Chryseobacterium indoltheticum*	Flavobacteria	2.36
S4	3.7 × 10^5^ ± 0.20	2.7 × 10^2^ ± 0.52	B6	*Chryseobacterium chaponense*	Flavobacteria	2.13
S5	6.7 × 10^6^ ± 0.46	6.2 × 10^3^ ± 0.72	B7	*Arthrobacter oxydans*	Actinobacteria	2.16
S6	1.5 × 10^6^ ± 0.31	9.4 × 10^3^ ± 0.33	B8	*Rhodococcus erythropolis*	Actinobacteria	2.14
S7	1.7 × 10^6^ ± 0.26	2.3 × 10^2^ ± 0.64	B9	*Arthrobacter arylaitensis*	Actinobacteria	2.23
S8	6.8 × 10^6^ ± 0.46	6.5 × 10^3^ ± 0.24	B10	*Bacillus megaterium*	Bacilli	2.11
S9	8.7 × 10^6^ ± 0.55	2.4 × 10^3^ ± 0.62	B11	*Lactobacillus plantarum*	Bacilli	2.17
S10	6.7 × 10^6^ ± 0.46	6.2 × 10^3^ ± 0.72	B12	*Bacillus weihenstephanensis*	Bacilli	2.18
S10	9.2 × 10^6^ ± 0.31	7.8 × 10^2^ ± 0.38	B13	*Rhodococcus fascians*	Actinobacteria	2.16
S10	6.7 × 10^6^ ± 0.46	6.2 × 10^3^ ± 0.72	B14	*Sphingomonas echinoides*	Alphaproteobacteria	2.19

^1^ APB, aerobic psychrophilic bacteria. ^2^ Mn-Ox_b_, manganese oxidizing bacteria. ^3^ Log (score) values computed by comparison of the peak list for an unknown isolate with the reference main spectral pattern in the BIOTYPER database (ranging from 0 to 3).

## Data Availability

Not applicable.

## Supplementary Materials

The following are available online at https://www.mdpi.com/article/10.3390/biology10101004/s1, Table S1: General soil characteristics. Table S2: Correlation values of bacterial growth kinetics and Mn(III/IV) content from 24 to 120 h of incubation at 4 °C, 15 °C and 30 °C. Figure S1: Correlation between ROS production and Mn(III/IV) production at 4 °C, 15 °C and 30 °C at 120 h of incubation in Mn-enriched broth. With *p* < 0.05 it is assumed that the correlation is statistically significant. *N* = 3.
