# Supplementary material for: Manganese-Oxidizing Antarctic Bacteria (Mn-Oxb) Release Reactive Oxygen Species (ROS) as Secondary Mn(II) Oxidation Mechanisms to Avoid Toxicity
Source: Biology (Basel). 2021 Oct 6;10(10):1004. doi: 10.3390/biology10101004 (PMC8533519; doi:10.3390/biology10101004)
Supplement: Supplementary file 1 [file biology-10-01004-s001.zip › biology-1387163-supplementary.pdf]

Supplementary materials

Manganese-Oxidizing Antarctic Bacteria (Mn-Oxb) Release Reactive Oxygen Species (ROS) as Secondary Mn (II) Oxidation Mechanisms to Avoid Toxicity

Ignacio Jofré [1], Francisco Matus [1,3,4] ,Daniela Mendoza [1,3], Francisco Nájera [1], Carolina Merino [1,2,3,*]

Table S1. General soil characteristics.

Code	C_{total}[1] % ± SD	C:N[2]	N_{total} % ± SD	Texture	Humidity	pH Water	Mn_d[4] G Kg^{-1} Soil	Mn_o[5] g kg^{-1} Soil	Mn_p[6] g kg-1 Soil
S1	0.01 ± 0.00	9.5	0.01 ± 0.004	LS	6.5	8	0.03 ± 0.004	0.04 ± 0.007	0.003 ± 0.001
S2	0.02 ± 0.00	6.1	0.02 ± 0.003	LS	8.8	8.67	0.07 ± 0.003	0.06 ± 0.001	0.003 ± 0.001
S3	0.10 ± 0.01	7.3	0.10 ± 0.006	S	7.5	7.25	0.03 ± 0	0.03 ± 0.013	0.004 ± 0
S4	0.05 ± 0.00	2.5	0.05 ± 0.004	LS	6.4	7.91	0.03 ± 0.001	0.04 ± 0.001	0.004 ± 0
S5	0.02 ± 0.01	5.3	0.02 ± 0.007	LS	6.5	8.1	0.03 ± 0.002	0.04 ± 0.002	0.004 ± 0
S6	0.03 ± 0.01	6.9	0.04 ± 0.007	LS	6.4	7.39	0.06 ± 0.008	0.06 ± 0.002	0.005 ± 0
S7	0.02 ± 0.01	7	0.02 ± 0.005	LS	7.4	8.18	0.03 ± 0	0.04 ± 0.001	0.007 ± 0
S8	0.04 ± 0.01	9.5	0.04 ± 0.005	SL	10	7.83	0.06 ± 0.003	0.08 ± 0.002	0.004 ± 0.001
S9	0.00 ± 0.00	120.3	0.01 ± 0.002	SL	4.3	9.15	0.06 ± 0.005	0.06 ± 0.004	0.007 ± 0.001
S10	3.21 ± 0.05	6.3	3.21 ± 0.054	L	1	6.61	0.01 ± 0	0.01 ± 0	0.01 ± 0

[1]Carbon total;
[2]Carbon-Nitrogen ratio;
[3]LS: Loamy Sand, SL: Sandy Loam and S: Sand
[4]Dithionite extractable Mn;
[5]Oxalate extractable Mn;
[6]Pyrophosphate extractable Mn;

Tables S2: Correlation values of bacterial growth kinetics and Mn (III/IV) content from 24 to 120h of incubation at 4°C, 15°C and 30°C.

		Growth (OD600nm) vs Mn (III/IV) content					
		**4°C**		**15°C**		**30°C**	
		r	P-value	r	P-value	r	P-value
B1	*M. esteraromaticum*	0.98	0.01	0.98	0.02	0.98	0.01
B2	*P. extremorientalis*	0.99	0.02	0.98	0.01	0.93	0.02
B3	*V. paradoxus*	0.94	0.01	0.94	0.01	0.88	0.01
B4	*A. psychrolactophilus*	0.99	0.02	0.97	0.01	0.99	0.01
B5	*C. indoltheticum*	0.93	0.02	0.94	0.02	0.94	0.02
B6	*C. chaponense*	0.95	0.01	0.95	0.01	0.96	0.01
B7	*P. oxydans*	0.97	0.01	0.91	0.03	0.99	0.01
B8	*R.erythropolis*	0.89	0.04	0.89	0.04	0.88	0.04
B9	*A. arylaitensis*	0.22	0.01	0.92	0.03	0.22	0.72
B10	*B. megaterium*	0.98	0.01	0.96	0.01	0.9	0.03
B11	*L. plantarum*	0.87	0.01	0.95	0.01	0.97	0.01
B12	*B. weihenstephanensis*	0.97	0.02	0.97	0.02	0.98	0.02
B13	*R. fascians*	0.97	0.01	0.91	0.03	0.91	0.03
B14	*S. echinoides*	0.96	0.01	0.96	0.01	0.99	0.01

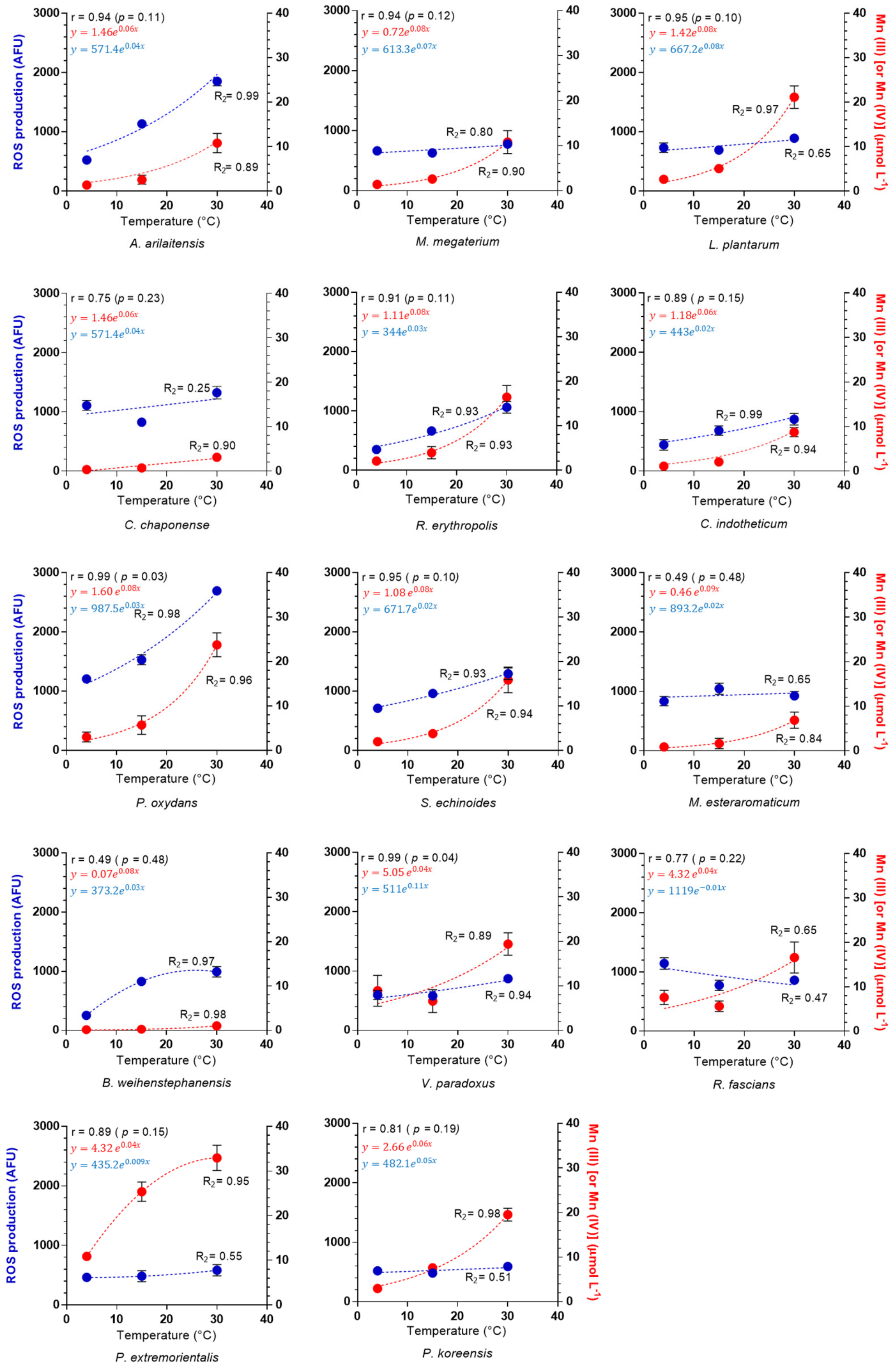

Figure S1: Correlation between ROS production and Mn (III/IV) production at 4 °C, 15 °C and 30 °C at 120 h of incubation in Mn-enriched broth. With $p < 0.05$ it is assumed that the correlation is statistically significant. N = 3.
